# Modification of Polyacrylonitrile Ultrafiltration Membranes to Enhance the Adsorption of Cations and Anions

**DOI:** 10.3390/membranes12060580

**Published:** 2022-05-31

**Authors:** Anthony Arvind Kishore Chand, Barbara Bajer, Erik S. Schneider, Tomi Mantel, Mathias Ernst, Volkan Filiz, Sarah Glass

**Affiliations:** 1 Institute of Membrane Research, Helmholtz-Zentrum Hereon, Max-Planck-Str. 1, 21502 Geesthacht, Germany; anthony.kishore.chand@tuhh.de (A.A.K.C.); barbara.bajer@hereon.de (B.B.); erik.schneider@hereon.de (E.S.S.); volkan.filiz@hereon.de (V.F.); 2 Institute for Water Resources and Water Supply (B-11), Hamburg University of Technology, Am Schwarzenberg-Campus 3E, 21073 Hamburg, Germany; tomi.mantel@tuhh.de (T.M.); mathias.ernst@tuhh.de (M.E.)

**Keywords:** membrane post-modification, PAN membranes, ion adsorption, water purification, adsorptive ultrafiltration membranes

## Abstract

Ion adsorbing ultrafiltration membranes provide an interesting possibility to remove toxic ions from water. Furthermore, it is also possible to recover valuable elements. In this work, we demonstrate two easy strategies to modify polyacrylonitrile membranes with anion and cation adsorbing groups. The membranes were modified to have positively charged amine groups or negatively charged carboxyl groups. The success of the reactions was confirmed using IR spectroscopy and zeta-potential measurements. The membranes carrying negatively charged groups provided a negative zeta-potential and had an isoelectric point at pH 3.6, while the membranes carrying positively charged groups had a positive zeta-potential in the analyzed pH range. Since only the surface of the polymer was modified, the pore size and permeance of the membranes were not drastically affected. The membranes prepared by both modification strategies had a pure water permeance higher than 1000 L/(m^2^ h bar) and a water contact angle of 44.3 and 57.2°, respectively. Therefore, the membranes can be operated at low pressures with reasonable flux. Additionally, SEM images showed that the membranes were still open-pored. Adsorption tests using a positively and a negatively charged dye as well as a toxic cation and an anion were performed to analyze the adsorption behavior. Both membranes were able to adsorb the oppositely charged dyes as well as the copper and chromate ions. Therefore, these membranes are good candidates to purify water streams containing hazardous ions.

## 1. Introduction

Safe drinking water is an essential resource. However, many regions in the world are facing serious challenges such as water scarcity and water pollution. Former studies estimated that by 2025, between one and two third of the world’s population will be living in water-stressed areas [1]. The driving forces for this are the rapid increase in population and climate change. Therefore, it is necessary to make the best use of the available water and eliminate contaminants as much as possible.

Some water constituents of concern are particles, pathogens, heavy metals such as oxyanions (chromate, arsenate [2]) or cations (lead, copper [3,4], mercury, zinc [5]), dissolved organic compounds [6], etc. Several of these ionic contaminants are related to serious diseases. Arsenic, for example, causes stomach pain, nausea, vomiting and cancer of the skin, bladder, lungs, kidney and many other organs [7,8,9].

Therefore, several technologies, such as ion exchange, membrane separation [10], adsorption [11], coagulation and flocculation, were reported in the past to remove toxic ionic contaminants from (waste)water [12]. Among these, hybrid systems combing two or more of the respective techniques are of special interest because they combine their advantages and can annihilate the drawbacks of each other. One method that gained attention in the past years is electrodialysis. Electrodialysis is a membrane separation technique that applies electric potential to remove ions from water. Former studies demonstrated the removal of heavy metal ions such as iron using this technique [13]. Another interesting hybrid technique is the combination of adsorption and membrane separation. Adsorptive membranes attained a lot of attention in the recent past because of their simplicity, effectiveness and versatility [14]. They combine high flow rates, low internal diffusion resistance and fast adsorption/desorption rates.

Membrane adsorbent technology in general, was developed in the 1980s by combining membranes with adsorbents, that bind molecules either by physical or chemical interactions. In general, chemical adsorption is favorable because the interactions are stronger and therefore, higher adsorption capacity can be achieved [15]. Adsorptive membranes can be made in various ways. Common preparation strategies are blending of two or more homopolymers, usage of copolymer-membranes, grafting of adsorptive molecules on the polymer membrane or dispersion of adsorbents in membranes (mixed matrix membranes) [14]. Additionally, the chemical post-modification of (polymeric) membranes is a powerful tool to prepare adsorptive membranes. Previous studies showed that membranes modified with nitrogen containing groups such as amines and imines showed promising anion adsorption behavior [16,17] as well as the ability to adsorb cations [18,19,20]. However, post-modification steps aiming for ion adsorbing membranes are commonly performed on membranes made from fiber mats or microfiltration membranes.

In this research, we present adsorptive ultrafiltration (UF) membranes prepared by post-modification of polyacrylonitrile (PAN) membranes. Ultrafiltration membranes are usually not capable of rejecting ions due to their pore size (10–100 nm). Contaminants in the sub-nanometer scale such as ions usually have to be filtered using nanofiltration (NF) or reverse osmosis (RO) membranes. However, NF and RO membranes need transmembrane pressures of >5 bar and require extensive post-treatment to become suitable for drinking water applications [21]. The applied pressure is mainly responsible for the energy consumed in membrane processes [22] and therefore, is one of the major issues that have to be solved in future sustainable membrane systems [23]. In this study, we aimed to prepare ion adsorbing membranes that can be operated at 1 bar and below. Thus, UF membranes were chemically modified by the introduction of functional groups usually used in ion exchange resins or adsorptive polymer fibers. Materials carrying these functional groups showed high adsorption capacities in past studies [17,24]. PAN membranes were used because they are commonly applied in several industries as ultrafiltration and microfiltration membranes. Usually, the membranes are formed by phase inversion processes. PAN membranes can be made from PAN purely [25] or as from PAN-containing co-polymer using other polymers such as poly(vinyl alcohol) [26] or polyacrylamide [27]. Therefore, they are interesting materials for membrane post-modification.

Ion exchange and adsorbing materials usually use counter ionic functional groups to adsorb cations and anions. This means that anions are adsorbed using positively charged groups (e.g., quartenary amines) [16] while cations are adsorbed using negatively charged groups (e.g., carbonyl groups) [28,29]. In this research, poylacrylonitrile was modified with the respective chemical groups. The success of the reactions was demonstrated using IR spectroscopy and zeta-potential measurements. Additionally, adsorption tests using charged organic dyes and ions showed that the modified membranes were able to adsorb charged water contaminants.

## 2. Materials and Methods

### 2.1. Materials

Sodium hydroxide and ethanol were purchased from Merck KGaA (Darmstadt, Germany). Sodium chloride, hydrochloric acid, ethylenediamine (EDA), methylene blue, orange II, 1-bromooctane and 1-bromobutane were bought from Sigma Aldrich (St. Louis, MI, USA). Methyl iodide from VWR International (Radnor, PA, USA). All chemicals were used without any further purification.

### 2.2. Preparation of PAN

The PAN membranes were prepared by non-solvent induced phase separation (NIPS) from a procedure as described by Scharnagl et al. [25]. In brief, 8 wt% PAN was dissolved in dimethylformamide (DMF) and γ-butyrolactone (GBL). Afterwards, the solution was coated on a non-woven polyphenylene sulfide support using a doctor blade with a gap height of 200 µm. The membranes were then drop-cast in water at room temperature, washed with water and dried. A SEM image of the cross-section of the freshly prepared pristine membrane is displayed in Figure S8.

### 2.3. Modification of PAN Using NaOH

**Scheme 1:**
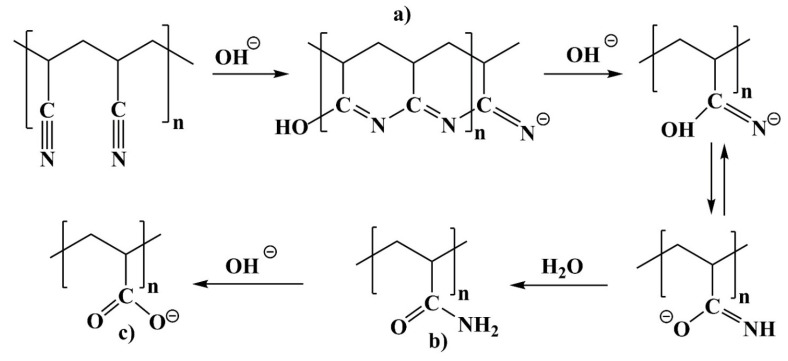
Reaction of PAN with NaOH with (intermediate) products: (**a**) Cyclic structure, (**b**) Amide and (**c**) carboxylic groups.

The reaction of PAN with NaOH at 75 °C is shown in Scheme 1. 75 mm diameter pristine PAN membranes were taken. 100 mL ethanol-water mixture (mole ratio—0.22:1) per membrane was used as the solvent. The reaction mixture containing the pristine PAN membranes and solvent was heated to 75 °C and stirred at 250 rpm under reflux conditions. On reaching 75 °C, NaOH solution was added using a dropping funnel. 4 mole NaOH per 1 mole PAN were used. The reaction was carried out for various durations as shown in Table 1. After the respective duration, the membranes were taken out of the mixture and washed three times for 30 min each with distilled water until a neutral pH was reached. The membranes were then dried under vacuum at 60 °C.

As shown in Table 1, abbreviations were used to denote the samples. The general abbreviation used to denote the PAN-NaOH membrane is PAN-NaOH-D where ‘PAN-NaOH’ refers to the reaction of PAN membrane with NaOH and ‘D’ represents the duration of the reaction in minutes.

### 2.4. Modification of PAN Using Ethylenediamine (EDA)

**Scheme 2:**
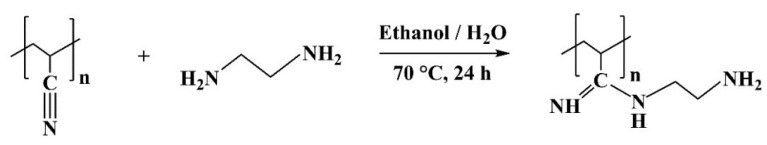
Reaction equation of PAN with ethylenediamine.

The reaction equation of PAN with EDA is shown in Scheme 2. The preparation of the PAN-EDA membrane was performed through a modified synthesis from El-Newehy [30] and Glass et al. [31]. Pristine PAN membranes of 75 mm diameter were used (0.38 g; 7.2 mmol monomer units). The pristine PAN membrane was placed in a flask and the solvent (100 mL ethanol and 20 mL distilled water) was added. Then a 10-fold molar excess of EDA (72 mmol) compared to PAN was added to the reaction mixture. The reaction mixture was then heated to 70 °C and stirred at 250 rpm. The reaction was carried out for 24 h. After the completion of the reaction, the PAN membranes were taken out and washed with ethanol for 30 min twice and then with distilled water (30 min). The washed membranes were then dried overnight at 60 °C under vacuum.

### 2.5. Quaternization of PAN-EDA

**Scheme 3:**
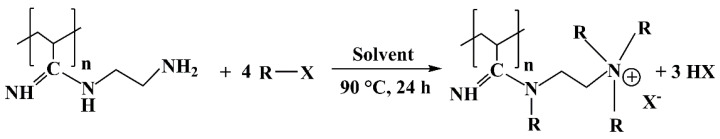
Quaternization reaction of PAN-EDA.

The general reaction scheme for the quaternization reaction of the PAN-EDA membrane is shown in Scheme 3. The alkyl halides (R-X) used were methyl iodide (CH_3_I), 1-bromobutane (1-C_4_H_9_Br) and 1-bromooctane (1-C_8_H_17_Br).

The mole ratio between PAN (7.2 mmol monomer units) and the alkyl halide was 1:5. 100 mL ethanol was used as the solvent. The PAN-EDA membranes were placed in a flask followed by the addition of the solvent and 36 mmol of the respective alkyl halide. The reaction mixture was then heated to 90 °C and stirred at 250 rpm for 24 h under reflux conditions. The quaternized-PAN-EDA membranes were taken out and washed twice for 30 min with fresh ethanol and then once with distilled water for 30 min. The washed membranes were then dried overnight in an oven at 60 °C under vacuum.

As seen in Table 2, abbreviations were used to denote the samples. E.g., PAN-EDA-MeI denotes the PAN-EDA membrane quaternized with methyl iodide. The general abbreviation used to denote quaternized-PAN-EDA membrane is ‘PAN-EDA-AH’ where AH represents the alkyl halide used for the quaternization reaction. The abbreviation of the alkyl halides (AH) is ‘MeI’ denotes methyl iodide, ‘BrBu’ represents 1-Bromobutane and ‘BrO’ represents 1-Bromooctane.

### 2.6. ATR-FTIR Analysis

The characterization method used to determine the presence of functional groups on the membrane surface was ATR-FTIR (attenuated total reflection Fourier-transform infrared spectroscopy). The measurement device used was ALPHA-P from Bruker (Billerica, MA, USA). The spectra were recorded between 4000 cm^−1^ and 400 cm^−1^ from the average of 32 scans at a resolution of 4 cm^−1^ using a diamond probe head.

### 2.7. Streaming Potential Measurements

The zeta potential is an indication of the surface charge of the membrane. It was measured using the SurPASS Eco 3 from Anton Paar (Graz, Austria). The streaming potential method was used to measure the zeta potential. The electrolyte solution used was 0.01 M NaCl solution and the pH was adjusted using 0.05 M NaOH and 0.05 M HCl. All solutions were prepared using ultrapure Milli-Q water. Before starting the zeta potential measurement, the membranes were rinsed multiple times with the electrolyte solution. The measurements were performed in the pH range 9 to 3. At each pH, the zeta potential was measured four times.

### 2.8. SEM Analysis

Scanning electron microscopy (SEM) was used to investigate the membrane morphology. SEM images were recorded on a Merlin SEM (Zeiss, Jena, Germany) at an accelerating voltage of 3 keV using an InLens secondary electron detector. Before measurement, the samples were dried under vacuum at 60 °C for 48–72 h and were sputter-coated with 1 nm platinum using a CCU-010 coating device (Safematic, Zizers, Switzerland). The pore size and porosity of the membrane surface were analyzed with the software IMS (Imagic Bildverarbeitung AG, Opfikon, Switzerland). The medium pore diameter (pore size) and the number of pores on the surface (surface porosity) were determined. Pores with an area smaller than 5 nm^2^ were excluded from the analysis. The values are given as the mean value ± standard deviation. In the case of pore size, anaverage of all pores was used.

### 2.9. Pure Water Permeability

The permeance measurement provides information on the pore size and the amount of pores on the membrane. Higher permeance indicates that there were more pores or larger pores. The permeance was measured using an inbuilt permeance measurement device in dead-end mode. It was measured on a circular piece of the membrane with a diameter of 2.0 cm corresponding to an active area (A) of 1.68 cm^2^. Ultrapure water was used to measure the permeance. The measurement was done multiple times to see if the results were reproducible. The permeance (J) was calculated using the following equation:

(1) $$J=\frac{∆V}{∆p\;\times ∆t\;\times A}$$

where ΔV represents the difference in volume, Δp was the transmembrane pressure (2 bar), Δt was the time interval (60 s) and A the area of membrane (1.68 cm^2^) respectively. The unit of permeance is given in L/(m^2^ h bar).

### 2.10. Water Contact Angle

The water contact angle (WCA) was measured to determine the hydrophilicity of the membranes. A water contact angle greater than 90° indicates a hydrophobic surface. This condition is due to poor wetting, poor adhesion and low solid surface free energy. A droplet with a contact angle lesser than 90° indicates a hydrophilic surface [32]. The WCA was measured using the sessile drop method with the Krüss drop shape analyser (Hamburg, Germany) and analyzed by the Krüss Advance software (DSA100, Krüss, Hamburg, Germany). The droplet used was distilled water with a volume of 2 μL. The measurement was performed under normal atmospheric conditions. The average of the left and the right contact angles was taken to determine the medium contact angle. The medium contact angle was measured four times for independent samples. The values are given as the mean value ± standard deviation.

### 2.11. Dye Adsorption Tests

The adsorption of two dyes (i.e., a negatively charged dye and a positively charged dye) was analyzed. Methylene blue was chosen as the positively charged dye and orange II was chosen as the negatively charged dye. A 5 µM solution of each dye was prepared separately. Thus, the solution contained 1.7 ± 0.1 mg/L methylene blue and 1.8 mg/L orange II, respectively. Membrane pieces with a diameter of 2.5 cm^2^ were used (active membrane surface 3.14 cm^2^).

Prior to the adsorption tests, water was filtered through the membranes at 1 bar transmembrane pressure for 1 h to avoid swelling during the measurements. Afterwards, the dye solutions were filtered through the membranes using an Amicon^®^ cell in dead-end mode (while stirring) at 1 bar transmembrane pressure. Samples of the permeate and the retentate were taken after 5, 15, 30, 45, 60, 90 and 120 min. Additionally, a sample of the feed solution was taken.

The concentration of the dye in the respective sample was determined using UV/VIS spectroscopy (GENESYS 10S spectrophotometer, Thermo Scientific, Waltham, MA, USA). The absorbance of methylene blue was measured at 665 nm and that of orange II was measured at 452 nm. The absorbance of each sample was compared with a previously measured calibration curve to calculate the concentration.

Three samples of each modified membrane were measured. The values are given as the mean value ± standard deviation.

### 2.12. Ion Adsorption Tests

The adsorption of two toxic ions—Cu^2+^ and CrO_4_^2−^—, that are commonly found in water streams was analyzed. Filtration experiments were carried out in dead-end mode using an Amicon^®^ filtration cell with an active membrane area of 28 cm^2^ (Model 8200, Merck Millipore, Darmstadt, Germany). The filtration feed contained either 5 µmol/L Cu^2+^ or 5 µmol/L CrO_4_^2−^. The filtration flux was kept constant (100 L/(m^2^ h)) in a fully automated pilot plant (Hydra, Convergence, Enschede, The Netherlands). The transmembrane pressure of the measurements was between 0.1 and 0.2 bar. The transmembrane pressure was automatically adjusted by the gear pump of the pilot plant. Permeate samples were collected every 20 min (Fraction Collector, Lambda Laboratory Instruments, Baar, Switzerland) and analyzed for Cu^2+^ and CrO_4_^2−^.

CrO_4_^2−^ concentrations were measured photometrically by applying the diphenylcarbazide method (Spectrophotometer DR5000, Hach Lange, Berlin, Germany) [33]. For this, 400 µL diphenylcarbazide solution and 400 µL of 2 M sulfuric acid were added to 20 mL of the samples. The absorbance of the solution was measured at a wavelength of 540 nm. The absorbance was then compared to a previously measured calibration curve. The concentration of Cu^2+^ was measured using a Spectroquant Copper Testkit (Merck KGaA, Darmstadt, Germany). The measurement was performed according to the instructions. Thus, 5 mL of the sample was mixed with reagent 1 (1 spoon) and reagent 2 (5 drops) and mixed for 5 min. The absorbance of the freshly prepared samples was measured at 605 nm and compared to a previously measured calibration curve.

## 3. Results

### 3.1. Modification of PAN Membranes by Intoduction of Cation Adsorbing Groups

**Figure 1:**
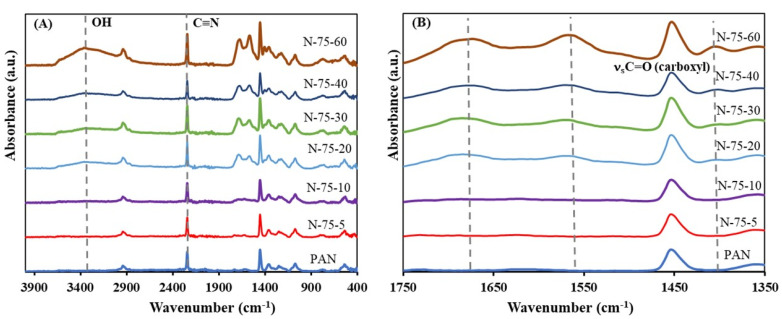
ATR-FTIR spectra of PAN-NaOH membranes modified for 5, 10, 20, 30, 40 and 60 min. (**A**) complete spectra and (**B**) spectral range of 1350 to 1750 cm^−1^. range from 1750–1350 cm^−1^.

The complete ATR-FTIR spectra of PAN-NaOH membranes heated to 75 °C are shown in Figure 1A). Pristine PAN membranes showed bands at 2940 cm^−1^ that corresponded to an asymmetric stretch of CH (ν-CH) in CH_2_ or CH_3_ groups, the band at 2242 cm^−1^ corresponded to the stretching vibration of the nitrile bond (ν-C≡N) and the band at 1452 cm^−1^ corresponds to the bending vibration of CH (δ-CH) in the CH_2_ groups [34,35].

A zoomed version of the spectra (1750 cm^−1^ to 1350 cm^−1^) is shown in Figure 1B. The figure displays the spectral range, where C=O and C=N bonds (such as amide and carboxylic groups formed during this reaction) show characteristic signals. As seen in Figure 1B, on performing the reaction at 75 °C for 5 min, there was a new band seen at 1618 cm^−1^. This band corresponds to a combination of C=C, C=N and NH in-plane bending of the ladder structure as shown in Scheme 1a [34]. The formation of the C=N band is an indication that the cyclisation of PAN occurred at 5 min. On increasing the duration of the reaction to 10 min, it was seen that the intensity of this band increased suggesting that the cyclisation reaction was still prominent. However, on increasing the duration to 20 min, it was noted that in addition to the band at 1618 cm^-1^, there were bands present at 3332 cm^−1^, 1670 cm^−1^, 1563 cm^−1^ and 1406 cm^−1^. The band at 3332 cm^−1^ indicated the stretching vibration of OH groups (νOH). The band at 1670 cm^−1^ corresponded to the stretching vibration of C=O (νC=O) in the amide group and the bands at 1563 cm^−1^ and 1406 cm^−1^ corresponded to the asymmetric (ν_as_C=O) and symmetric vibration of C=O (ν_s_C=O) in carboxylate group, respectively [34]. Therefore, at 20 min, the cyclic structure of PAN was converted to amide (Scheme 1b) and carboxylic groups (Scheme 1c). On increasing the reaction duration to 30 min, the bands corresponding to the amide and carboxyl groups were still present while the band corresponding to C=N was decreasing. With a further increase in the reaction duration to 40 min and 60 min, the C=N band was absent indicating that the cyclic structure of PAN was completely converted to amide and carboxylic groups. Furthermore, with an increase in reaction duration, it was noted that there was an increase in the ratio of the band intensities between the carboxylic and amide groups. This suggests that the amide groups were further converted to carboxylic groups as the duration of the reaction increased (see Scheme 1b,c).

**Figure 2:**
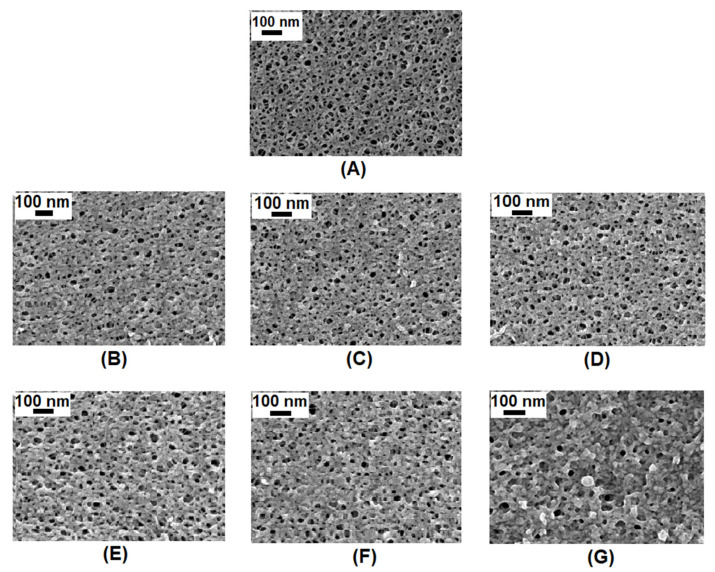
SEM images of the surface of (**A**) pristine PAN membrane and PAN-NaOH modified for (**B**) 5 min, (**C**) 10 min, (**D**) 20 min, (**E**) 30 min, (**F**) 40 min and (**G**) 60 min.

SEM analysis (Figure 2) was performed to determine if there were changes in pore size and surface porosity of the membranes occurring during the reaction. There was a slight change in the structure of the membrane with an increase in reaction duration (5 min to 60 min). The SEM images were used to calculate the pore size and the porosity of the membrane surface. Table 3 shows that the porosity of the pristine membrane was higher than the porosity of the modified membranes. The change in surface porosity is due to a slight change in themorphology of the membrane, which is clearly visible in Figure 2. Additionally, there was a marginal decrease in the surface porosity of the membranes as the duration was increased from 5 min to 60 min while the pore size did not change drastically. All membranes had a mean pore size of 11 to 12 nm.

Additionally, the WCA of the PAN-NaOH membranes are shown in Table 3. It was observed that all PAN-NaOH membranes, except for PAN-NaOH-60, had a similar WCA as the pristine PAN membrane (about 45°) and were hydrophilic. PAN-NAOH-60 had a slightly lower WCA of 37.4°. This was due to the increased amount of the hydrophilic carboxyl and amide groups on the surface of the membrane [36].

**Figure 3:**
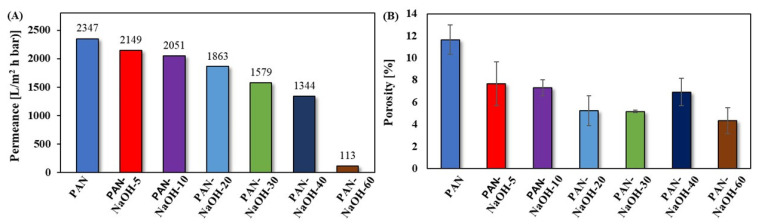
(**A**) Initial permeance compared to the (**B**) porosity of PAN-NaOH membranes modified for 5, 10, 20, 30, 40 and 60 min.

The water permeance of the membranes (Figure 3A) was measured to see if the change in morphology affected the membrane performance. As seen in Figure 3A, the permeance decreased with an increase in the duration of the reaction (i.e.,) from 5 min to 60 min.

The initial water permeance (Figure 3A) of the pristine PAN membrane was 2350 L/(m^2^ h bar). It is necessary for the pristine PAN membrane to have a high water permeance since the aim was to have membranes that can be operated at low transmembrane pressures. In order to achieve this high water permeance, the PAN membranes were prepared from a polymer solution with a low concentration (8 wt%). The initial water permeance of the modified PAN-NaOH membranes decreased slightly with an increase in reaction duration. It decreased from 2150 L/(m^2^ h bar) for 5 min reaction time to 1340 L/(m^2^ h bar) for 40 min. For 60 min reaction, the permeance decreased drastically to 113 L/(m^2^ h bar) indicating that the pores were smaller or partially blocked. One reason for the decrease in permeance could be the formation of the carboxyl group (COO^−^) on the membrane surface. According to Muthumareeswaran et al., the formation of COO^−^ on the surface leads to a decrease in the porosity [10]. The reason for the decreased porosity (Figure 3B) was a swelling of the membrane caused by the alkaline treatment, which was reported before [37]. The swelling of the membrane caused a disappearance of small pores. As a result, the porosity decreased. Due to thedecrease in porosity, less water can pass through the membrane. Furthermore, there was a larger decrease in the water permeance compared to the porosity due to the presence of carboxylic groups, causing additional swelling, when the membrane comesin contact with water. This can be explained by the higher hydrophilicity of carboxylic groups. Therefore, the decrease in water permeance was larger than the decrease in surface porosity in a dry state.

**Figure 4:**
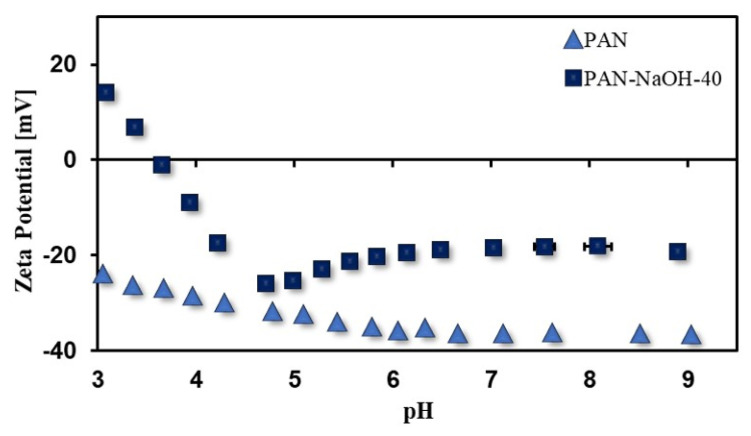
Zeta potential (pH 9 to 3) of PAN-NaOH-40 membranes (reaction at 75 °C for 40 min).

According to the ATR-IR spectra, the PAN-NaOH-40 membrane had the highest degree of modification as well as a high water permeance, and therefore as a next step, the zeta potential of this membrane was analyzed. The zeta potential graphs of pristine PAN and PAN-NaOH-40 membranes are shown in Figure 4. It could be observed that the zeta potential of pristine PAN membrane was completely negative in the pH range from 9 to 3. It ranged from −37 mV at pH 9 to −22 mV at pH 3. No IEP was observed in this pH range. The surface charge was negative due to the adsorption of hydroxyl ions on the membrane surface [38]. The surface charge of the modified membranes increased. The PAN-NaOH-40 membrane had a surface charge ranging from −19 mV to 20 mV with an IEP at pH 3.6. The IEP was present due to the protonation of COO^-^ to COOH. A similar IEP was also reported by Muthumareeswaran et al. [10].

### 3.2. Modification of PAN Membranes by Introduction of Anion Adsorbing Groups

**Figure 5:**
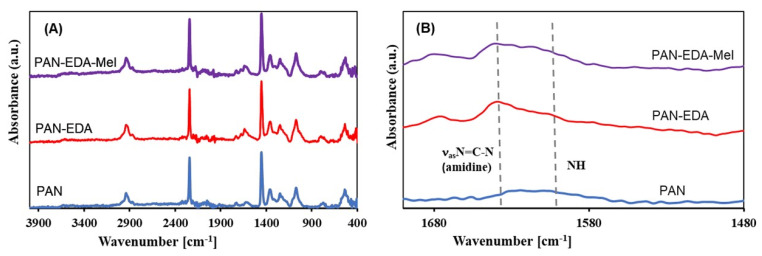
ATR-FTIR spectra of pristine PAN, PAN-EDA and PAN-EDA-MeI membranes (**A**) complete spectra and (**B**) spectral range from 1700 to 1480 cm^−1^.

The ATR-FTIR spectra of the pristine PAN, PAN-EDA and PAN-EDA-MeI membranes are shown in Figure 5A. Pristine PAN spectrum was similar to Section 3.1. At a first glance, there was no major difference between the spectra of PAN-EDA and pristine PAN membranes. However, on zooming in the range of 1480–1750 cm^−1^ (Figure 5B), there were two new bands visible for PAN-EDA, that were not present in the pristine PAN membrane. On the one hand, there was one signal at 1650 cm^−1^ corresponding to the stretching vibration of the amidine group (N=C-N). The presence of the amidine group suggests the success of the amination reaction [31]. On the other hand, a small band at 1577 cm^−1^ was seen which corresponds to the stretching vibration of the NH group [18]. The signal at 1650 cm^−1^ (N=C-N group) was still visible for the PAN-EDA-MeI membrane. However, the N-H bond is only present in the PAN-EDA modification (see Scheme 2 and Scheme 3) therefore, the IR band at 1577 cm^−1^ disappeared after the second modification step. Similar results were obtained, when performing the quaternization with 1-bromobutane and 1-bromoctane (see Figure S2).

**Figure 6:**
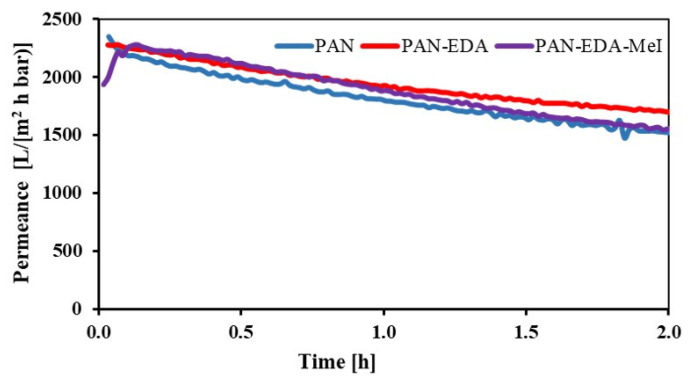
Permeance of pristine PAN, PAN-EDA and PAN-EDA-MeI membranes.

The permeance of the PAN-EDA membrane (Figure 6) suggested that the structure of the membrane did not vary much since it had a similar trend as the pristine PAN membrane. The initial permeances of pristine PAN, PAN-EDA and PAN-EDA-MeI membranes were 2350 L/(m^2^ h bar). The same was observed for PAN-EDA-BrBu and PAN-EDA-BrO (see Figure 3). As already mentioned, the permeance decreased with time due to the swelling of the membrane in water.

**Figure 7:**
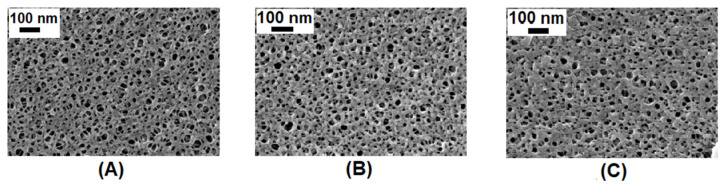
SEM images of the surface of (**A**) pristine PAN, (**B**) PAN-EDA, (**C**) PAN-EDA-MeI membranes.

Furthermore, the SEM images (Figure 7) showed no visible changes in the morphology between the pristine PAN, PAN-EDA and PAN-EDA-MeI membranes. This suggests that there was no big change in the structure of the membrane after both modification steps. The structure of PAN-EDA-BrBu and PAN-EDA-BrO membranes (see Figure S4) was also similar to the pristine PAN and PAN-EDA membranes.

The WCA of pristine PAN, PAN-EDA and the quaternized-PAN-EDA-MeI are shown in Table 4. The pristine PAN membrane and the PAN-EDA membrane had WCA of 46.8 ± 1.9° and 42.1 ± 5.5°, respectively, indicating that the membranes were hydrophilic. The presence of the hydrophilic NH groups on the PAN-EDA membranes decreased the contact angle of the membranes slightly but not significantly [39]. On the other hand, the quaternized-PAN-EDA-MeI membrane was slightly more hydrophobic compared to the PAN-EDA and pristine PAN membranes. This was due to the alkylation of the hydrophilic NH group in PAN-EDA by methyl-substituents as seen in Scheme 3. The same effect was observed for PAN-EDA-BrBu and PAN-EDA-BrO membranes (see Figure S3).

**Figure 8:**
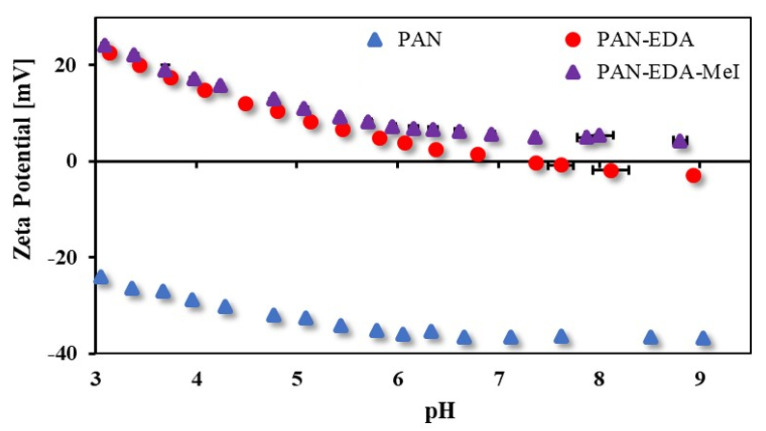
Zeta potential (pH 9 to 3) of pristine PAN, PAN-EDA and PAN-EDA-MeI membranes.

The zeta potential results of pristine PAN, PAN-EDA and quaternised PAN-EDA-MeI membranes are shown in Figure 8. As seen from the zeta potential, the reaction of PAN with EDA increased the surface charge of the membrane. The PAN-EDA membrane had a zeta potential value ranging from −4 mV to 24 mV with an IEP at pH 7.2. Therefore, from the zeta potential, it was confirmed that the reaction of PAN with EDA occurred. Furthermore, the zeta potential of the quaternized-PAN-EDA-MeI membrane was completely positive in the entire pH range from 9 to 3. There was no IEP observed since the quaternary amines cannot be deprotonated. The same effect was observed with PAN-EDA-BrBu and PAN-EDA-BrO membranes (see Figure S6). Combined with the IR spectra, this showed that the quaternization reaction was successful.

### 3.3. Ion Adsorption of Modified PAN Membranes

The adsorption behavior of the negatively charged PAN-NaOH-40 and the positively charged PAN-EDA-MeI was analyzed. First, the adsorption measurements were performed in a dead-end mode using a positively charged dye (methylene blue) and a negatively charged one (orange II). The chemical structures of both dyes are shown in Figure S7. Organic dyes such as the ones used in this study are 1–2 nm in size [40,41,42]. Compared to this, the pore size of the PAN membranes was about 12 nm (see Table 3 and Table 4). Therefore, rejection by size exclusion was not possible here but the main retention mechanism was based on electrostatic adsorption.

**Figure 9:**
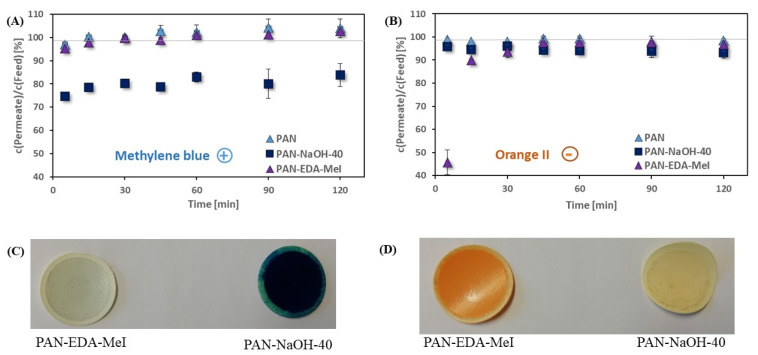
Adsorption of (**A**) positively charged methylene blue and (**B**) negatively charged orange II on PAN, PAN-NaOH-40 and PAN-EDA-MeI (grey lines display the feed concentration) as well as photos of the modified membranes after (C) methylene blue adsorption and (**D**) orange II adsorption experiments.

The dye adsorption experiments (Figure 9) showed that both modifications led to an improved adsorption behavior compared to the pristine PAN membrane. The negatively charged PAN-NaOH-40 adsorbed methylene blue (Figure 9A). Therefore, the concentration in the permeate decreased compared to the concentration in the feed solution. The negatively charged membranes were able to adsorb 25.3 ± 1.4% of the dissolved methylene blue initially. Even after 2 h, the membranes were still adsorbing 16.2 ± 5.0%. The positively charged membranes and the pristine PAN membranes were not adsorbing any of the methylene blue. Contrary to that, the negatively charged dye was adsorbed only by the positively charged PAN-EDA-MeI (Figure 9B). In the beginning, the positively charged membranes adsorbed high amounts of the dye, decreasing the concentration of orange II in the permeate by 52.3 ± 5.3%. Compared to Figure 9A, the adsorption capacity was faster and after 45 min the membrane was not able to adsorb more dye. However, the PAN-EDA-MeI membranes adsorbed more orange II than the pristine membrane and the negatively charged PAN-NaOH-40, which were not adsorbing the dye at all.

The change in adsorption behavior was even more obvious when the membranes were investigated after the dye adsorption experiments. In Figure 9C, both modified membranes after the adsorption of methylene blue are shown. The negatively charged PAN-NaOH-40 membrane strongly adsorbed the positive dye. Therefore, after the adsorption experiment, the membrane appeared to have a dark blue color The positively charged PAN-EDA-MeI membrane was only slightly colored because it did not adsorb the dye. The contrary was seen for the negatively charged orange II (Figure 9D). Here, the positively charged PAN-EDA-MeI had an intense orange color after the adsorption of the negatively charged dye.

**Figure 10:**
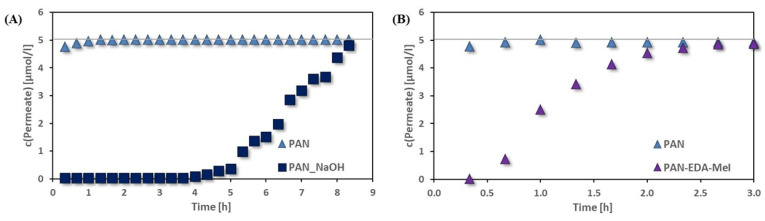
Adsorption of (**A**) Cu^2+^ cation on negatively charged PAN-NaOH-40 and (**B**) CrO_4_^2−^ anions on PAN-EDA-MeI compared to pristine PAN. (Grey lines display the feed concentration.)

Once the membranes showed potential to adsorb oppositely charged substances, adsorption experiments using a toxic heavy metal cation (Cu^2+^) and a toxic anion (CrO_4_^2−^) were performed. The results of these adsorption experiments are shown in Figure 10. The negatively charged PAN-NaOH-40 membrane (Figure 10A) adsorbed Cu^2+^ from the feed almost completely for 4 h. After 4 h the concentration of Cu^2+^ in the permeate slowly increased. After 8 h, the adsorption capacity of the membranes was reached, and therefore, the concentration of Cu^2+^ in the feed and the permeate was the same. The positively charged membrane PAN-EDA-MeI (Figure 10B) was able to adsorb CrO_4_^2−^. However, the adsorption capacity of the membrane was attained much faster than the negatively charged membrane. The chromate concentration in the permeate was zero for only 20 min, after which, the concentration of CrO_4_^2−^ increased. After 2 h, the PAN-EDA-MeI membrane did not adsorb any further chromate.

Nevertheless, both membranes were able to adsorb the oppositely charged ions while the pristine membranes did not adsorb any ions. Also, both membranes showed high adsorption capacities (1.61 mg/g copper on PAN-NaOH-40 and 0.47 mg/g chromate on PAN-EDA-MeI). Therefore, both modifications were successful and generated ion adsorbing groups on the membranes.

## 4. Discussion

In this study, PAN membranes were successfully modified such that either anion- or cation adsorbing groups were present on the surface. These groups enable the adsorption of hazardous cations and anions, on the surface respectively. Two modification strategies were carried out. The first strategy (i.e.,) hydrolysis using NaOH was synthesized for the adsorption of hazardous cations and the second strategy (i.e.,) quaternisation of PAN-EDA membrane was prepared for the adsorption of hazardous anions. The success of both modification strategies was confirmed using IR spectroscopy and zeta potential measurements. The best modification results were obtained for the PAN membrane modified with NaOH for 40 min at 75 °C and the PAN membrane modified with ethylene diamine and methyl iodine for 24 h.

The PAN membranes modified using NaOH had carboxyl and amide groups present on the surface. Polymers modified with these groups showed high cation-adsorption properties in previous studies and can adsorb toxic cations such as copper(II) and lead(II). For example, Deng et al. [11], showed that modified PAN fibers adsorbed 2.8 to 12.4 mg/g copper depending on the adsorption conditions. The membranes prepared in this study had a comparable high adsorption capacity of 1.61 mg/g. The quaternized-PAN-EDA membranes had positively charged amine groups on the surface, shown by the positive zeta potential in the entire pH range studied. Since, the surface charge of the quaternized-PAN-EDA membranes was positive; this membrane can be used to adsorb hazardous anions such as chromate. The chromate adsorption capacity of the positively charged membranes was lesser (0.47 mg/g) compared to the cation adsorbing membranes.

The adsorption of either negatively or positively charged small molecules was shown by dead-end-adsorption experiments using charged dyes. While the unmodified membranes were not able to adsorb any dye, the modified membranes were able to absorb the oppositely charged dye. Neither adsorption nor retention of the similarly charged dyes was observed. This showed that the successfully introduced ion adsorbing groups were indeed able to adsorb oppositely charged small molecules and ions. Additionally, the adsorption of a toxic cation (copper) and a toxic anion (chromate) was analyzed. While the pristine membranes did not adsorb or reject the ions, the charged membranes were able to adsorb the oppositely charged ion. Therefore, the purification of water from toxic ions as well as the harvesting of valuable metal ions using these membranes is possible.

In comparison to other polymeric materials used for membranes, PAN can be modified easily. The nitrile groups present in PAN allow several chemical reactions such as hydrolysis, cyclization and amination [43,44]. Both synthesis strategies shown here were carried out in non-toxic solvents (namely, ethanol and water). Compared to the post-modification reactions of other membrane materials such as PVDF or PES that need toxic solvents, several modification steps or pre-activation, the modifications presented here were easier and more environment friendly [45,46,47].

Additionally, membranes prepared by both modification strategies had permeance greater than 1000 L/(m^2^ h bar). Therefore, the membranes can be operated at pressures lower than 0.2 bar with reasonable flux (>100 L/(m^2^ h)). The adsorption and permeation experiments in this study were performed at transmembrane pressures of 1 to 2 bar without breakage of the membrane. Therefore, the membranes were mechanically stable enough to be used at low pressures. Operation at low pressures is advantageous as it consumes low energy as well as, the hydrostatic pressure is sufficient. The operation at low pressures or with no external pressure at all is of special interest; because water-stressed areas are often also areas with regular energy shortages. Therefore, the membranes presented here can be applied in the purification of wastewater streams and in decentralized ultra-low pressure water treatment systems.

## 5. Conclusions

In conclusion, two easy modification strategies to prepare ultrafiltration membranes carrying anion- and cation adsorbing groups were synthesized. The rejection of the molecules and ions by size exclusion was not possible due to the pore size of the membranes. Nevertheless, the success of the modifications as well as their ability to adsorb charged small molecules and ions was shown. Both membranes were able to adsorb ions at transmembrane pressures smaller than 0.2 bar. In the next steps, the adsorption of toxic cations and anions will be improved by optimizing the NaOH modified PAN membrane and the quaternized-PAN-EDA membranes. Additionally, the modification strategies used here will be tested on hollow-fiber membranes as well. Once the modification is optimized, application-oriented studies (including experiments on reuse and desorption) will be conducted.

## Figures and Tables

**Table 1:** Reaction conditions of PAN-NaOH membranes reacted at 75 °C for different durations.

Duration of Reaction (min)	Abbreviation
5	PAN-NaOH-5
10	PAN-NaOH-10
20	PAN-NaOH-20
30	PAN-NaOH-30
40	PAN-NaOH-40
60	PAN-NaOH-60

**Table 2:** Quaternization of PAN-EDA membranes with alkyl halides.

Alkyl Halide	Abbreviation
CH_3_I	PAN-EDA-MeI
1-C_4_H_9_Br	PAN-EDA-BrBu
1-C_8_H_17_Br	PAN-EDA-BrO

**Table 3:** Water contact angle, pore size and porosity of PAN-NaOH membranes modified for 5, 10, 20, 30, 40 and 60 min. Given are mean values ± standard deviation; n = number of measurements.

	PAN-Pristine	PAN-NaOH-5	PAN-NaOH-10	PAN-NaOH-20	PAN-NaOH-30	PAN-NaOH-40	PAN-NaOH-60
Pore size (nm)	12.6 ± 6.7	11.8 ± 6.1	11.4 ± 6.1	11.7 ± 6.2	12.8 ± 7.5	11.9 ± 6.2	11.5 ± 8.4
	(n = 5216)	(n = 3949)	(n = 4037)	(n = 2715)	(n = 1087)	(n = 3510)	(n = 1003)
Surface porosity (%)	11.7 ± 1.3	7.7 ± 2.0	7.3 ± 0.7	5.3 ± 1.3	5.2 ± 0.1	6.9 ± 1.2	4.3 ± 1.2
	(n = 6)	(n = 6)	(n = 6)	(n = 6)	(n = 3)	(n = 6)	(n = 3)
Water contact angle (°)	46.3 ± 1.9	43.0 ± 5.9	47.4 ± 1.8	44.3 ± 3.2	43.6 ± 2.8	44.1 ± 4.8	37.4 ± 5.5
	(n = 3)	(n = 3)	(n = 3)	(n = 3)	(n = 3)	(n = 3)	(n = 3)

**Table 4:** Water contact angle, pore size and porosity of PAN-EDA and PAN EDA-MeI membranes. Given are mean values ± standard deviation; n = number of measurements.

	PAN-Pristine	PAN-EDA	PAN-EDA-MeI
Pore size (nm)	12.6 ± 6.7	12.2 ± 6.1	12.2 ± 6.2
	(n = 5216)	(n = 4186)	(n = 4129)
Surface porosity (%)	11.7 ± 1.3	9.3 ± 0.6	9.2 ± 1.2
	(n = 6)	(n = 6)	(n = 6)
Water contact angle (°)	46.8 ± 1.9	42.1 ± 5.4	57.2 ± 5.5
	(n = 3)	(n = 3)	(n = 3)

## Data Availability

The data presented in this study are available on request from the corresponding author.

## Supplementary Materials

The following are available online at https://www.mdpi.com/article/10.3390/membranes12060580/s1, Figure S1: Pure water permeance of pristine PAN membranes and PAN NaOH membranes modified for 5, 10, 20, 30, 40 and 60 min measured for 2 h, Figure S2: ATR FTIR spectra of pristine PAN, PAN EDA and PAN-EDA-1BrBu and PAN-EDA-BrO membranes (a) complete spectra and (b) spectral range from 1700–1480 cm^−1^, Figure S3: Permeance of pristine PAN, PAN EDA, PAN-EDA-1BrBu and PAN-EDA-BrO membranes, Figure S4: SEM analysis of (a) pristine PAN, (b) PAN EDA-1BrBu, (c) PAN EDA BrO membranes, Figure S5: Water contact angle of PAN-EDA, PAN EDA-BrBu and PAN-EDA-BrO membranes, Figure S6: Zeta potential (pH 9 to 3) of PAN EDA and PAN-EDA-1BrBu and PAN-EDA-BrO membranes. Figure S7: Chemical structure of (a) methylene blue and (b) orange II. Figure S8: SEM images of the cross section of the pristine PAN membrane.

## References

[1] Arnell N.W. (2004). Climate change and global water resources: Sres emissions and socio-economic scenarios. Glob. Environ. Change.

[2] National Toxicology Program (2008). Toxicology and carcinogenesis studies of sodium dichromate dihydrate (cas no. 7789-12-0) in f344/n rats and b6c3f1 mice (drinking water studies). Natl. Toxicol. Program Tech. Rep. Ser..

[3] Velizarov S., Matos C., Oehmen A., Serra S., Reis M., Crespo J. (2008). Removal of inorganic charged micropollutants from drinking water supplies by hybrid ion exchange membrane processes. Desalination.

[4] Brown K.G., Ross G.L. (2002). Arsenic, drinking water, and health: A position paper of the american council on science and health. Regul. Toxicol. Pharmacol..

[5] Căprărescu S., Modrogan C., Purcar V., Dăncilă A.M., Orbuleț O.D. (2021). Study of polyvinyl alcohol-sio2 nanoparticles polymeric membrane in wastewater treatment containing zinc ions. Polymers.

[6] Dayarathne H.N.P., Angove M.J., Aryal R., Abuel-Naga H., Mainali B. (2021). Removal of natural organic matter from source water: Review on coagulants, dual coagulation, alternative coagulants, and mechanisms. J. Water Process Eng..

[7] Sharma S., Bhattacharya A. (2017). Drinking water contamination and treatment techniques. Appl. Water Sci..

[8] Salnikow K., Zhitkovich A. (2008). Genetic and epigenetic mechanisms in metal carcinogenesis and cocarcinogenesis: Nickel, arsenic, and chromium. Chem. Res. Toxicol..

[9] Zhitkovich A. (2011). Chromium in drinking water: Sources, metabolism, and cancer risks. Chem. Res. Toxicol..

[10] Muthumareeswaran M.R., Alhoshan M., Agarwal G.P. (2017). Ultrafiltration membrane for effective removal of chromium ions from potable water. Sci. Rep..

[11] Deng S., Bai R., Chen J.P. (2003). Behaviors and mechanisms of copper adsorption on hydrolyzed polyacrylonitrile fibers. J. Colloid Interface Sci..

[12] Nicomel N.R., Leus K., Folens K., Van Der Voort P., Du Laing G. (2016). Technologies for arsenic removal from water: Current status and future perspectives. Int. J. Environ. Res. Public Health.

[13] Căprărescu S., Zgârian R.G., Tihan G.T., Purcar V., Eftimie Totu E., Modrogan C., Chiriac A.-L., Nicolae C.A. (2020). Biopolymeric membrane enriched with chitosan and silver for metallic ions removal. Polymers.

[14] Hao S., Jia Z., Wen J., Li S., Peng W., Huang R., Xu X. (2021). Progress in adsorptive membranes for separation—A review. Sep. Purif. Technol..

[15] Khulbe K.C., Matsuura T. (2018). Removal of heavy metals and pollutants by membrane adsorption techniques. Appl. Water Sci..

[16] Ma Y., Zhang B., Ma H., Yu M., Li L., Li J. (2016). Electrospun nanofibrous polyethylenimine mat: A potential adsorbent for the removal of chromate and arsenate from drinking water. RSC Adv..

[17] Kampalanonwat P., Supaphol P. (2010). Preparation and adsorption behavior of aminated electrospun polyacrylonitrile nanofiber mats for heavy metal ion removal. ACS Appl. Mater. Interfaces.

[18] Aung K.T., Hong S.-H., Park S.-J., Lee C.-G. (2020). Removal of cu(ii) from aqueous solutions using amine-doped polyacrylonitrile fibers. Appl. Sci..

[19] Yang D., Li L., Chen B., Shi S., Nie J., Ma G. (2019). Functionalized chitosan electrospun nanofiber membranes for heavy-metal removal. Polymer.

[20] Wang Y., Wang B., Wang Q., Di J., Miao S., Yu J. (2019). Amino-functionalized porous nanofibrous membranes for simultaneous removal of oil and heavy-metal ions from wastewater. ACS Appl. Mater. Interfaces.

[21] Singh R., Hankins N.P., Hankins N.P., Singh R. (2016). Introduction to membrane processes for water treatment. Emerging Membrane Technology for Sustainable Water Treatment.

[22] Saleh T.A., Gupta V.K., Saleh T.A., Gupta V.K. (2016). Chapter 1—An overview of membrane science and technology. Nanomaterial and Polymer Membranes.

[23] Obotey Ezugbe E., Rathilal S. (2020). Membrane technologies in wastewater treatment: A review. Membranes.

[24] Zheng W., Hu J., Han Z., Wang Z., Zheng Z., Langer J., Economy J. (2015). Synthesis of porous carbon fibers with strong anion exchange functional groups. Chem. Commun..

[25] Scharnagl N., Buschatz H. (2001). Polyacrylonitrile (pan) membranes for ultra- and microfiltration. Desalination.

[26] Sandu T., Sârbu A., Damian C.M., Marin A., Vulpe S., Budinova T., Tsyntsarski B., Yardim M.F., Sirkecioglu A. (2014). Preparation and characterization of membranes obtained from blends of acrylonitrile copolymers with poly(vinyl alcohol). J. Appl. Polym. Sci..

[27] Fei Z.-D., Wan L.-S., Wang W.-M., Zhong M.-Q., Xu Z.-K. (2013). Thermo-responsive polyacrylonitrile membranes prepared with poly(acrylonitrile-g-isopropylacrylamide) as an additive. J. Membr. Sci..

[28] Zheng G., Ye H., Zhang Y., Li H., Lin L., Ding X. (2015). Removal of heavy metal in drinking water resource with cation-exchange resins (type 110-h) mixed pes membrane adsorbents. J. Hazard. Toxic Radioact. Waste.

[29] Golubenko D.V., Voropaeva D.Y., Yaroslavtsev A.B. (2020). Cation-exchange membranes with sulfonylimide groups showing a high ionic conductivity in water/organic amide mixed systems. Mater. Lett..

[30] El-Newehy M.H., Alamri A., Al-Deyab S.S. (2014). Optimization of amine-terminated polyacrylonitrile synthesis and characterization. Arab. J. Chem..

[31] Glass S., Mantel T., Appold M., Sen S., Usman M., Ernst M., Filiz V. (2021). Amine-terminated pan membranes as anion-adsorber materials. Chem. Ing. Tech..

[32] Guy O.J., Walker K.-A.D., Saddow S.E. (2016). Chapter 4—Graphene functionalization for biosensor applications. Silicon Carbide Biotechnology.

[33] Gröhlich A., Langer M., Mitrakas M., Zouboulis A., Katsoyiannis I., Ernst M. (2017). Effect of organic matter on cr(vi) removal from groundwaters by fe(ii) reductive precipitation for groundwater treatment. Water.

[34] Jin S.Y., Kim M.H., Jeong Y.G., Yoon Y.I., Park W.H. (2017). Effect of alkaline hydrolysis on cyclization reaction of pan nanofibers. Mater. Des..

[35] Karacan I., Erdogan G. (2012). The influence of thermal stabilization stage on the molecular structure of polyacrylonitrile fibers prior to the carbonization stage. Fibers Polym..

[36] Nemani S.K., Annavarapu R.K., Mohammadian B., Raiyan A., Heil J., Haque M.A., Abdelaal A., Sojoudi H. (2018). Surface modification of polymers: Methods and applications. Adv. Mater. Interfaces.

[37] Boyraz E., Yalcinkaya F. (2021). Hydrophilic surface-modified pan nanofibrous membranes for efficient oil-water emulsion separation. Polymers.

[38] Zimmermann R., Freudenberg U., Schweiß R., Küttner D., Werner C. (2010). Hydroxide and hydronium ion adsorption—A survey. Curr. Opin. Colloid Interface Sci..

[39] Allcock H.R. (1996). Water-soluble polyphosphazenes and their hydrogels. Hydrophilic Polymers.

[40] Glass S., Rüdiger T., Griebel J., Abel B., Schulze A. (2018). Uptake and release of photosensitizers in a hydrogel for applications in photodynamic therapy: The impact of structural parameters on intrapolymer transport dynamics. RSC Adv..

[41] Feng X., Kawabata K., Kaufman G., Elimelech M., Osuji C.O. (2017). Highly selective vertically aligned nanopores in sustainably derived polymer membranes by molecular templating. ACS Nano.

[42] Zhang Z., Rahman M.M., Abetz C., Höhme A.-L., Sperling E., Abetz V. (2020). Chemically tailored multifunctional asymmetric isoporous triblock terpolymer membranes for selective transport. Adv. Mater..

[43] Sogawa H., Wang C.-G., Monjiyama S., Akae Y., Takata T. (2021). Aliphatic ditopic nitrile n-oxide crosslinker: Synthesis, chemical stability, and catalyst-free crosslinking reactions. Polymer.

[44] Sruthi P.R., Anas S. (2020). An overview of synthetic modification of nitrile group in polymers and applications. J. Polym. Sci..

[45] Wu L., Sun J., Tong F. (2014). Surface modification of a pvdf membrane by cross-linked collagen. RSC Adv..

[46] Han H., Dai R., Wang Z. (2020). Fabrication of high-performance thin-film composite nanofiltration membrane by dynamic calcium-carboxyl intra-bridging during post-treatment. Membranes.

[47] Hao S., Geng Y., Jia Z. (2018). Uv pre-activation/thermal initiated grafting of caffeic acid on pvdf for preparation of adsorptive membranes for cesium. React. Funct. Polym..

