# Supplementary material for: Modification of Polyacrylonitrile Ultrafiltration Membranes to Enhance the Adsorption of Cations and Anions
Source: Membranes (Basel). 2022 May 31;12(6):580. doi: 10.3390/membranes12060580 (PMC9228498; doi:10.3390/membranes12060580)
Supplement: Supplementary file 1 [file membranes-12-00580-s001.zip › membranes-1725145-supplementary.pdf]

membranes

MDPI

Supplementary Materials

Modification of Polyacrylonitrile Ultrafiltration Membranes to Enhance the Adsorption of Cations and Anions

Anthony Arvind Kishore Chand [1,2], Barbara Bajer [1], Erik S. Schneider [1], Tomi Mantel [2], Mathias Ernst [2], Volkan Filiz [1] and Sarah Glass [1,*]

[1] Institute of Membrane Research, Helmholtz-Zentrum Hereon, Max-Planck-Str. 1, 21502 Geesthacht, Germany; anthony.kishore.chand@tuhh.de (A.A.K.C.); barbara.bajer@hereon.de (B.B.); erik.schneider@hereon.de (E.S.S.); volkan.filiz@hereon.de (V.F.)

[2] Institute for Water Resources and Water Supply (B-11), Hamburg University of Technology, Am Schwarzenberg-Campus 3E, 21073 Hamburg, Germany; tomi.mantel@tuhh.de (T.M.); mathias.ernst@tuhh.de (M.E.)

* Correspondence: sarah.glass@hereon.de

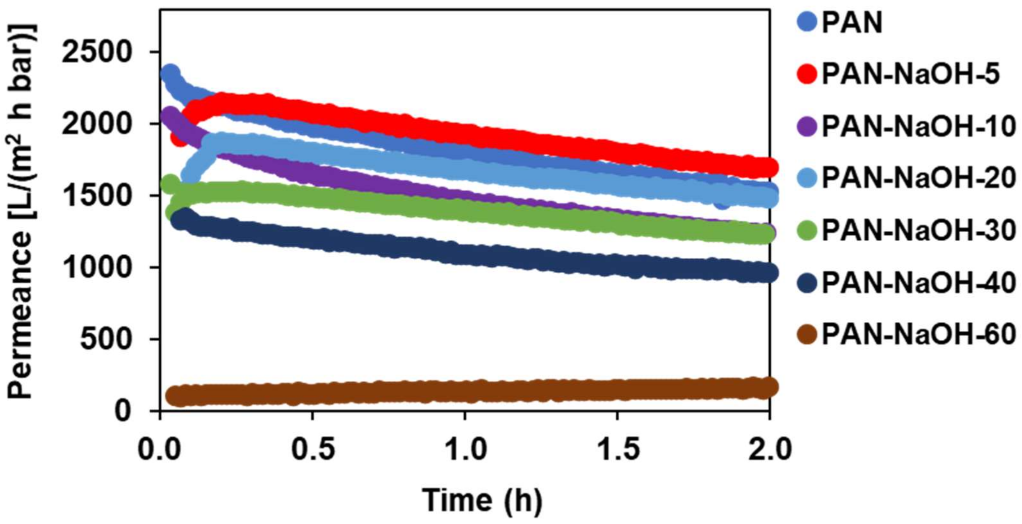

Figure S1. Pure water permeance of pristine PAN membranes and PAN-NaOH membranes modified for 5, 10, 20, 30, 40 and 60 min measured for 2 h.

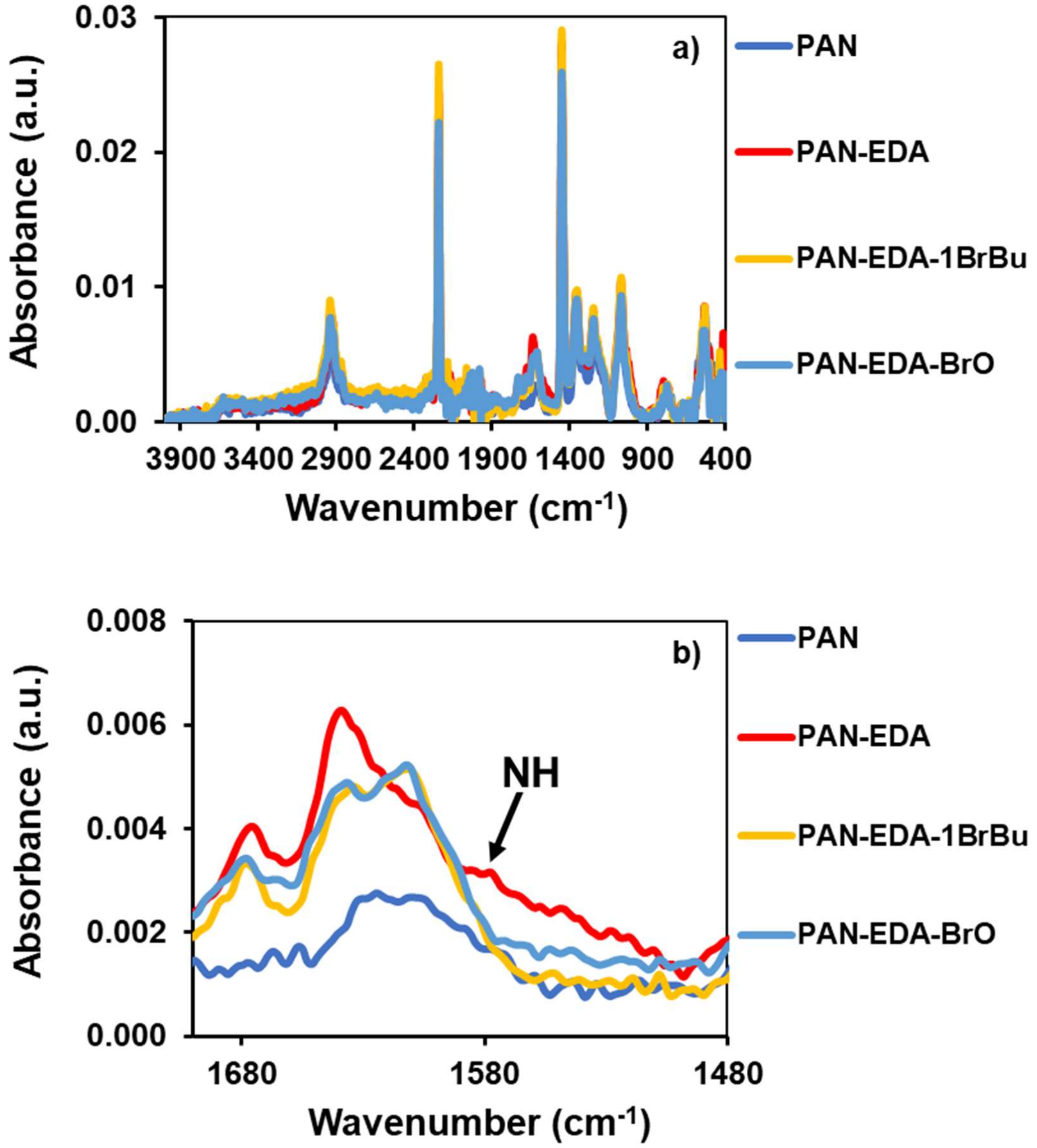

Figure S2. ATR-FTIR spectra of pristine PAN, PAN-EDA and PAN-EDA-1BrBu and PAN-EDA-BrO membranes a) complete spectra and b) spectral range from 1700-1480 cm^{-1}.

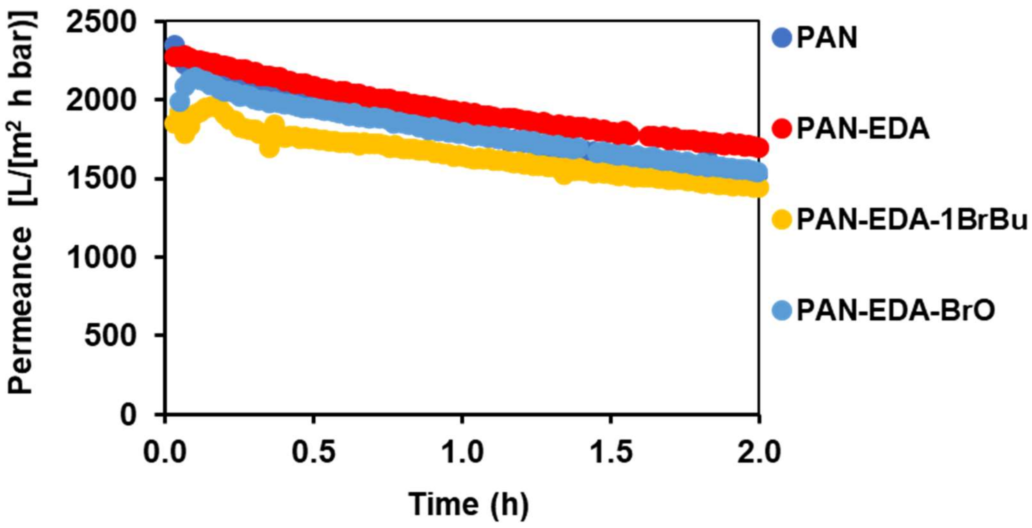

Figure S3. Permeance of pristine PAN, PAN-EDA, PAN-EDA-1BrBu and PAN-EDA-BrO membranes.

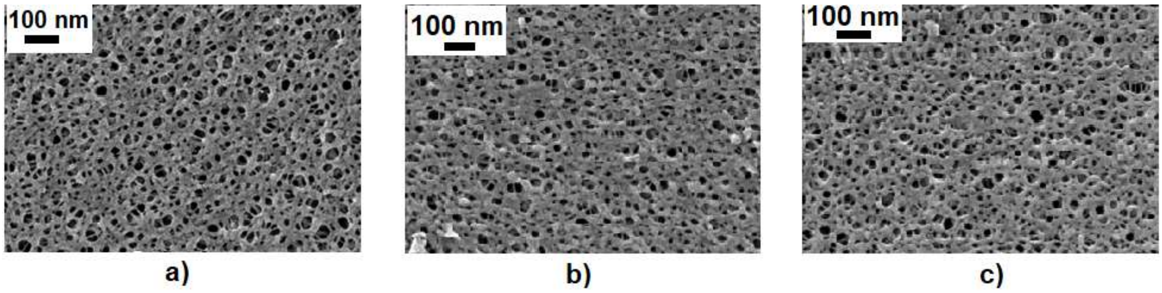

Figure S4. SEM analysis of a) pristine PAN, b) PAN-EDA-1BrBu, c) PAN-EDA-BrO membranes.

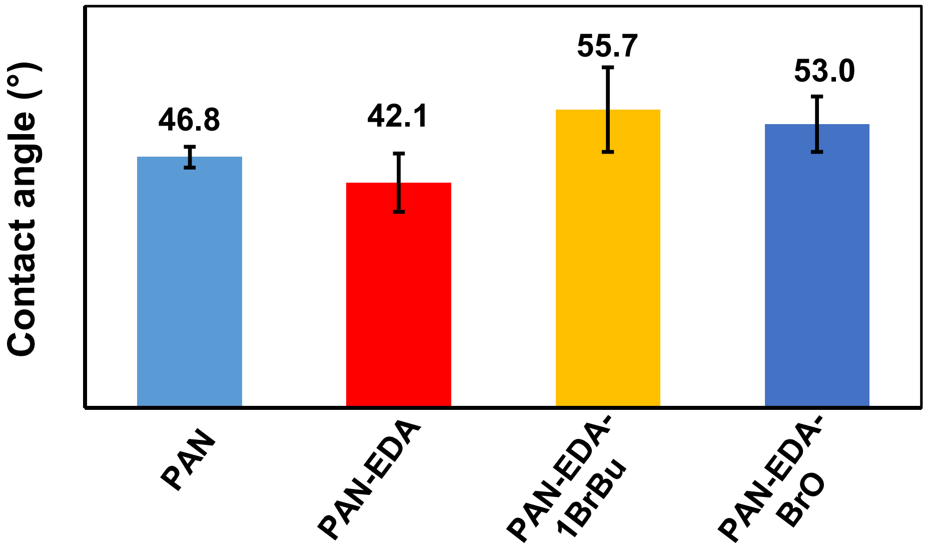

Figure S5. Water contact angle of PAN-EDA, PAN-EDA-1BrBu and PAN-EDA-BrO membranes.

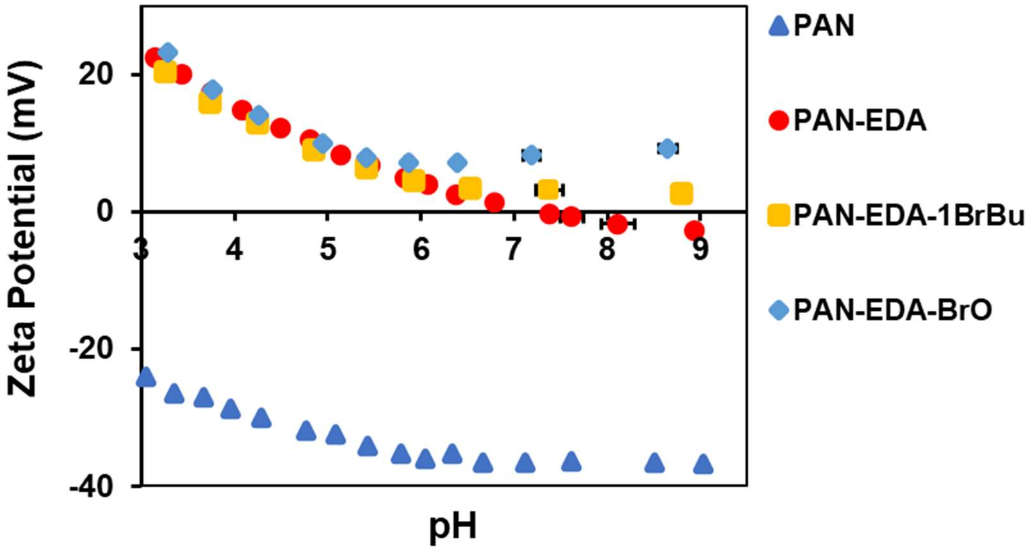

Figure S6. Zeta potential (pH 9 to 3) of PAN-EDA and PAN-EDA-1BrBu and PAN-EDA-BrO membranes.

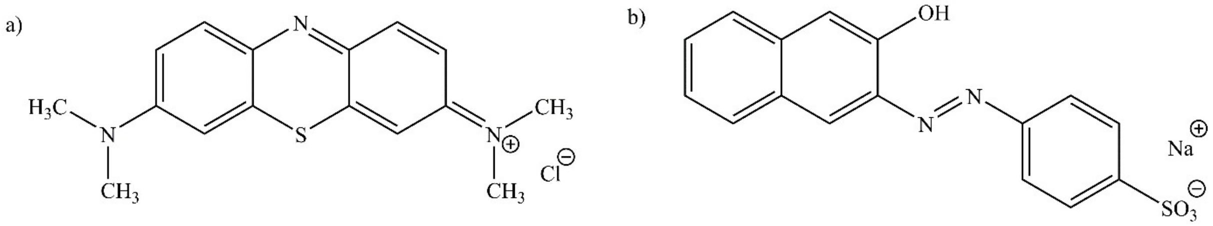

Figure S7. Chemical structure of a) methylene blue and b) orange II.

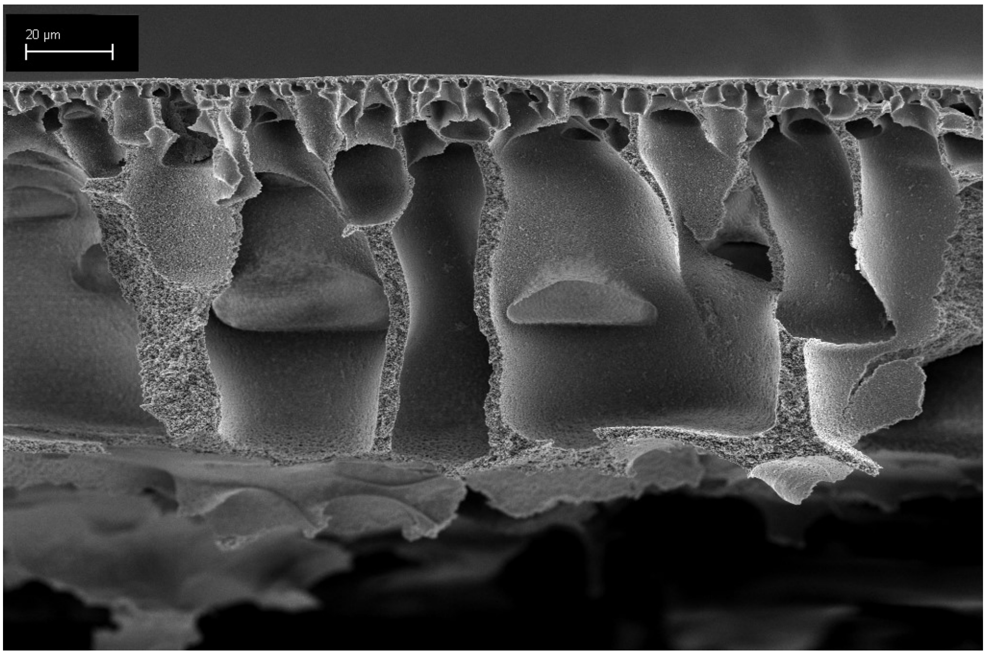

Figure S8. SEM images of the cross section of the pristine PAN membrane.
